# Debates in allergy medicine: baked egg and milk do not accelerate tolerance to egg and milk

**DOI:** 10.1186/s40413-015-0090-z

**Published:** 2016-01-26

**Authors:** Thanh D. Dang, Rachel L. Peters, Katrina J. Allen

**Affiliations:** Centre of Food and Allergy Research, Murdoch Childrens Research Institute, Melbourne, Australia; Department of Allergy and Clinical Immunology, The Royal Children’s Hospital, Melbourne, Australia; Department of Gastroenterology and Clinical Nutrition, The Royal Children’s Hospital, Melbourne, Australia; Department of Paediatrics, University of Melbourne, Parkville, Australia; Institute of Inflammation and Repair, University of Manchester, Manchester, UK; Murdoch Childrens Research Institute, The Royal Children’s Hospital, Parkville, Australia

**Keywords:** Food allergy, Egg allergy, Milk allergy, Baked, Tolerance, Immunotherapy, IgE, Component resolved diagnostics

## Abstract

There is emerging evidence that children with egg and cow’s milk allergy who can tolerate these allergens cooked in baked goods are more likely to develop tolerance. As a result a hypothesis has arisen that exposure to egg and milk in baked goods may hasten tolerance development; however, it is unclear whether children who develop tolerance do so because they have ingested low levels of egg or milk in baked products. An alternative explanation for the improved prognosis in those who can tolerate food allergens in the baked form is that tolerance to egg and milk in baked goods is simply an indicator of a phenotype that is less likely to be persistent. We discuss the role that the baked egg or milk allergy phenotype plays on predicting tolerance development and suggest that it is the phenotype of the disease rather than exposure to altered allergens that is the strongest predictor of tolerance development.

## Background

Egg and milk allergy are common in childhood as is development of tolerance to these two foods. There is great debate about why tolerance develops more commonly to these foods while allergy to peanut, tree nuts and shellfish are more likely to be lifelong. Both egg and milk in baked goods are less allergenic than the semi-cooked or uncooked form due to the conformational change in the allergen epitope associated with cooking at high temperatures [1]. In fact, the majority of children allergic to egg or milk in its raw form are able to tolerate egg and milk in baked goods. In addition to the altered nature of the food allergen in baked goods, the dose of egg and cow’s milk ingested in a serving of baked goods is usually lower than when eaten in a raw form. There is emerging evidence that children with egg and cow’s milk allergy who can tolerate these allergens cooked in baked goods are more likely to develop tolerance than those who are reactive to the allergen in its baked form [2, 3]. As a result a hypothesis has arisen that exposure to egg and milk in baked goods may hasten tolerance development. However it is unclear whether the differential rates of tolerance development is due to the ingestion of modified egg allergen in baked egg products or simply due to different clinical phenotypes of egg allergy.

## Egg and milk allergy are common

IgE-mediated raw egg and cow’s milk allergy present as the most common food allergies in young infants. A recent meta-analysis on the prevalence of food allergy estimated that egg allergy affects 0.5–2.5 % of young children in the western world [4]. However, the exact prevalence is difficult to ascertain, largely due to the differences between cohorts and the definition of allergy. In a recent population study of 1-year old infants with challenge proven outcomes, the prevalence of raw egg allergy was as high as 8.9 % [5]. The aforementioned meta-analysis also reported that the prevalence of self-reported cow’s milk allergy ranged from 1.2 to 17 % while the prevalence of challenge-confirmed milk allergy ranged from 0 to 3 % [4]. In a large population-based food allergy study of 1-year old infants, parent-reported adverse reaction to cow’s milk was 6.1 % (95 % CI, 5.1–7.0), however, only 2.7 % (95 % CI, 2.1–3.4) had a reaction consistent with symptoms of IgE-mediated food allergy [5].

Symptoms usually present rapidly and can manifest as mild urticaria, nausea, abdominal pain and/or vomiting, to more severe manifestations including anaphylaxis [6]. The current therapy for food allergy is strict avoidance in all dietary forms [7] however as egg and milk are versatile ingredients used for cooking in many cultures, as well as a wide range of manufactured food products, dietary avoidance can be difficult [8, 9]. Recent developments suggest that there are different phenotypes of egg and milk allergy based on the individual’s ability to tolerate the allergen in its baked form. Therefore strict dietary avoidance may not apply to all phenotypes of allergy.

## Are there different phenotypes for egg and milk allergy?

It has been demonstrated that amongst children allergic to egg and milk that up to 80 % can ingest small amounts in baked goods without reaction [2, 10, 11] with similar levels of tolerance to baked cow’s milk reported. Emerging evidence suggests that antibody response to subcomponents of each allergen can strongly predict the likelihood of baked egg or cow’s milk tolerance. As such, there are two distinct phenotypes of cow’s milk and egg allergy, those allergic who are tolerant to the baked form and those that are not, with little evidence to suggest that there is any association between these phenotypes with the severity of clinical reactions [12].

Egg white contains more than 20 proteins, a mixture of both allergenic and non-allergenic proteins. Despite Gal d 2 being the most abundant protein in egg white by weight (54 % of protein), Gal d 1 makes up 11 % and is the predominant egg allergen. There has been much debate over whether Gal d 2, as the major egg allergen, is immune dominant and responsible for causing the majority of clinically adverse reactions following egg ingestion. Three small studies have found that Gal d 1 levels were more accurate in predicting raw egg allergy compared to egg white sIgE and that higher levels of Gal d 1 are also associated with persistent egg white allergy [13–15]. While it was suggested that the lower the Gal d 1-sIgE concentration, the higher the probability of tolerance to baked eggs [14, 15], Tan et al*.*, Lemon-Mule et al*.,* and Bartnikas et al*.* did not find that Gal d 1 was a good predictor of tolerance to baked egg [13, 16, 17].

**Fig. 1:**
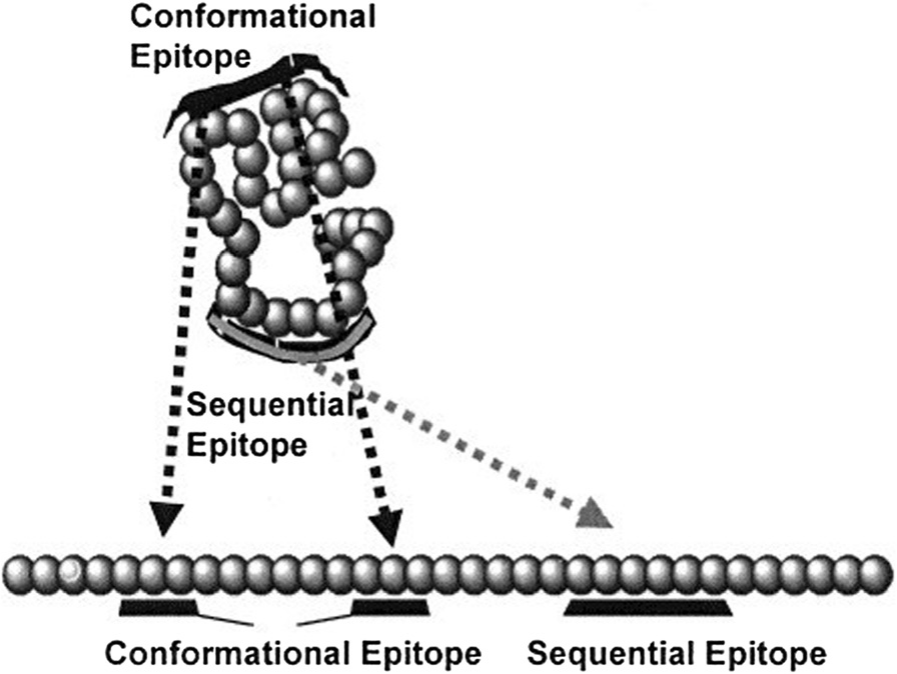
Conformational epitopes and sequential epitopes: conformational epitopes are destroyed when the shape of a protein is altered by cooking whereas sequential epitopes are not [40]

Approximately 70–80 % children who are allergic to egg in its raw or lightly-cooked form are able to tolerate egg in baked foods, such as cakes and biscuits [3, 5, 11, 18, 19]. Baking egg may decrease its allergenicity by destroying conformational epitopes or blocking epitope access through formation of a food matrix with wheat. The protein epitopes in egg can be sequential (linear comprising several amino acids in a row) or conformational (coiled or part of the shape of the protein) (Fig. 1). Extensive heating of egg induces changes in the conformational structure of the epitope, effectively destroying it, and thus reducing its allergenicity. Children who produce IgE antibodies that predominately recognise conformational epitopes can therefore ingest baked egg without reaction. In addition, baking the egg protein with wheat flour forms a food matrix, which also reduces the allergenicity of the protein by affecting the digestibility of the proteins or making the IgE binding sites less accessible [1, 20–22]. Several reports now suggest that these phenotypes of allergy distinguished by baked egg reactivity have an impact on the natural history of egg allergy, however the mechanism of this pathway remains unclear [3, 13, 23].

Similarly to egg, many cow’s milk allergic patients may be unnecessarily be avoiding cow’s milk, with up to 75 % of milk allergic patients found to be tolerant to baked-milk products such as muffins and waffles [2]. Cow’s milk represents an abundant source of allergens which, depending on the allergen sensitized to, may result in a different clinical phenotype. The most abundant milk allergens are caseins (Bos d 8), which makes up about 75–80 % of all milk allergens. Other less dominant milk allergens include whey proteins such as α-lactalbumin (Bos d 4, 5 %), β-lactoglobulin (Bos d 5, 10 %), and lactoferrin (Bos d lactoferrin) [24]. While D’urbano et al. found that Bos d 8 levels were most accurate in predicting milk allergy [25], it has been shown that persistent cow’s milk allergic patients are sensitised to more milk allergens with increased epitope diversity compared to baked milk tolerant patients [26, 27], highlighting the importance of allergen preparation and exposure.

## Effect of preparation on allergenicity

Both egg and cow’s milk are complex allergenic sources, the allergenicity of which can be modified by baking with other food ingredients such as wheat (and other grains containing gluten) in addition to the effect of heating during cooking [22, 28]. The conformation of most of these allergenic proteins changes during the cooking process with evidence of variation depending on whether boiled alone or baked in a food matrix with wheat and rice [29, 30]. Kato et al*.* previously showed that solubility of Ovomucoid (Gal d 1) decreased when egg was mixed with wheat flour and wheat gluten, suggesting that Gal d 1 forms insoluble complexes with gluten, potentially leading to decreased digestibility [22]. Rendering the protein insoluble could explain why such high levels of sIgE to Gal d 1 are needed to predict reactivity to baked egg in baked egg allergic children compared to children tolerating baked and lightly cooked egg [13]. Caseins (Bos D 8) are the most stable milk allergen, resisting heat treatment even after 120 min of boiling [28]. The effect of cooking milk proteins in a food matrix was highlighted recently when proteins extracted from muffins and waffles showed that Bos D 8 was still present after cooking in the wheat and rice matrix [28]. In contrast, whey proteins such as Bos d 4 and Bos d 5 were denatured within 30 min of heat treatment, and no longer present in the waffle and muffin. The ability of both Gal d 1 and Bos d 8 to survive heat treatment may explain their importance in determining persistent allergy compared to those that produce IgE antibodies to the other major allergens such as Ovalbumin (Gal d 2) and Bos d 4. Patients with antibodies to the latter are more likely to have good prognostic outcomes.

## Development of tolerance to egg and cow’s milk is common

Egg allergy has an excellent prognosis with reports in the past showing that 50 % of children with egg allergy will develop tolerance by 4 years of age and up to 80 % resolves by school-age [31]. Two recent studies have confirmed these findings. In the HealthNuts study it was demonstrated that 50 % of infants with challenge-confirmed egg allergy at 12-months had developed tolerance at 2 years of age [10]. The Consortium of Food Allergy Research (CoFAR) followed 213 children with egg allergy and found that 49 % developed tolerance at a median age of 72 months [32]. A comparison of the study characteristics and results are presented in Table 1. HealthNuts followed a subgroup of egg allergic children recruited from a population-based food allergy study and confirmed egg allergy at both baseline and follow-up with formal food challenges, whereas CoFAR recruited infants from clinics with a diagnosis of egg allergy on the basis of OFC or clinical history and sensitisation, and tolerance at follow up was determined by either OFC or home introduction. HealthNuts demonstrated that egg allergic infants who were baked egg tolerant were 5 times more likely to develop tolerance than those who were also baked egg allergic. Those who ingested baked egg goods more frequently appeared to have the best prognosis although it is difficult to know whether tolerance occurs because of the higher ingestion or despite this.

**Table 1:** Comparison of HealthNuts and Consortium of Food Allergy Research studies on the natural history of egg allergy [10, 32]

	CoFAR	HealthNuts
Baseline		
Sample size	213	140
Age range baseline	3-15 months (38 % >12 m)	12 months
Population	Subgroup of multi-centre observational study which recruited infants with a history of milk or egg allergy deemed at high risk of peanut allergy.	Subgroup of longitudinal population based cohort
Definition of egg allergy at baseline	Positive OFC; or previous reaction and egg sensitisation (SPT > = 3 mm and/ or sIgE > = 0.35 kU/L); or eczema flare following egg ingestion and sIgE > 2 (95 % PPV)	Positive OFC and egg sensititsation (SPT > = 2 mm and/ or sIgE > = 0.35 kU/L)
Gender (male)	70 %	61 %
OFC at baseline	Some (but not specified how many) 13 % diagnosed based on eczema flare	All, irrespective of SPT
Baked egg allergy phenotype	Parent-report of ingestion with or without reaction 6 months after recruitment	Baked egg OFC offered to all participants (parent-report in those who declined, 16 % of total)
Eczema at baseline	92 %	63 %
SPT at baseline	<5(24 %)	> = 4 mm 55 %
	> = 5 (76 %)	
sIgE at baseline	<2 (37 %)	> = 1.7 48 %
	2-10 (34 %)	
	> = 10 (29 %)	
OFC / reaction symptoms at baseline	Eczema flare 13 %	Skin symptoms 79 %
	Skin symptoms 44 %	Other systems 21 %
	Other systems 44 %	
Other food allergy	52 %	25 %
Follow-up		
Determination of egg allergy resolution	OFC (45 %) or home introduction (55 %)	OFC irrespective of SPT (100 %)
Resolution	49 %	47 %
Age resolution	72 months	27 months
Resolution among baked egg tolerant phenotype	71 %	54 %
Resolution among baked egg allergic phenotype	45 %	13 %
Resolution stratified by baked egg ingestion	No ingestion (45 %)	Frequent ingestion (61 %)
	Ingestion with reaction (57 %)	Infrequent ingestion (41 %)
	Ingestion without reaction (71 %)	No ingestion (17 %)
SPT as a predictor of outcome	<5 vs >10 HR 1.995 95 % CI 1.23-3.24	> = 4 mm OR 3.34 95 % CI 1.52-7.38, p 0.003
	5- < 10 vs >10 HR 0.860 95 % CI 0.55-1.35	
sIgE as a predictor of outcome	<2.0 vs >10 HR 3.874 95 % CI 2.25–6.66	> = 1.7kU/L OR 29.46 95 % CI 8.86–97.92 p < 0.001
	2–10 vs >10 HR 2.064 95 % CI1.19–3.59	
Other predictors of outcome	Severity of baseline eczema egg reaction class, sex, IgG4,	Baked egg allergy and frequency of baked egg ingestion
Other variables considered, not predictive	Baseline age, race, breastfeeding, other food allergy, asthma or rhinitis	Eczema, other food allergies, OFC symptoms and dose, FLG

*FLG* filaggrin, *HR* hazards ratio, *OFC* oral food challenge, *OR* odds ratio, *sIgE* specific IgE, *SPT* skin prick test

Milk allergy also generally presents with a good prognosis, however, there have been many studies with conflicting results on the resolution rates [33–37]. Bishop et al. found in a general paediatric population that 56 % of infants resolved their milk allergy by 4 years of age [34]. In contrast, a retrospective study of subjects referred to tertiary care centre published in 2007 by Skripak et al. found that the rates of resolution are slowing over time, with a large proportion of children persisting with disease into adolescence [33]. They found that only 19 % of milk allergic children resolved their allergy by 4 years of age, and 42 % by 8 years of age. Although these changes might be occuring, it is likely that differences in  study design and patient selection is contributing to differences in reported results. General population studies are more likely to demonstrate earlier resolution [34, 38] compared to studies of tertiary referral populations [33, 35, 36]. More recently, CoFAR followed 244 milk allergic children for 66 months and found that 50 % developed tolerance to milk at 5 years, falling in between previously published results [37]. However they did not characterize baked milk consumption extensively, and it remains unclear whether the ingestion of milk in baked goods influenced the resolution of milk allergy.

## What is the evidence that eating baked egg induces tolerance

It is controversial whether egg allergic children should strictly avoid all forms of egg, or if regular ingestion of baked egg will either delay or hasten the resolution of egg allergy. The impact of egg allergy phenotype on the natural history of egg allergy was first proposed by Konstantinou et al*.* in a report of 94 children who were either egg allergic (n = 55) or sensitised but had not ingested egg (n = 39). Children underwent OFC to baked egg and 93 % were tolerant [23]. Tolerant children were instructed to consume cake every day for six months, with a cumulative increase in egg protein. After 6 months, children underwent another OFC to boiled egg white and 95 % passed the OFC leading the authors to conclude that baked egg consumption may affect the natural course of egg allergy. However, these results should be interpreted with caution as there was no comparison group, that is, the rate of resolution was not reported in children who were baked egg reactive or baked egg tolerant but continued to avoid baked egg. It must also be noted that nearly half the participants were egg sensitised but had never ingested egg, so they may not have had true egg allergy. This may be reflected in the unusually high rate of presumed resolution reported [23].

In another study, 117 children and adults with documented egg allergy on the basis of clinical history or sIgE levels (>2 kU/L if < 2 years of age or sIgE > 7 kU/L if > 2 years of age), underwent OFC to baked egg [13]. 74 % of participants were tolerant to baked egg and their allergy to regular egg was either confirmed or assumed on the basis of sIgE > 95 % PPVs, leaving 64 egg allergic baked egg tolerant participants. These participants were instructed to consume baked egg 1–3 times per day and were evaluated at 3, 6 and 12 months; 18 participants withdrew from the study and were not included at follow-up. In the first 3 months of follow-up, egg white SPT significantly reduced and ovalbumin and ovomucoid-specific IgG4 levels increased, immunological changes that are frequently observed in the development of tolerance to egg. These changes were maintained until follow-up ceased at 12 months and led the authors to propose that ingestion of baked egg may affect the natural course of egg allergy. Unfortunately, this study was small and unable to recruit a comparison group of baked egg tolerant children who continued to avoid baked egg. It is therefore difficult to ascertain whether these changes are a result of baked egg ingestion or simply immunological changes that would have otherwise occurred in the natural course of egg allergy [13].

In a follow-up study, this cohort was retrospectively matched on the basis of age, sex and sIgE to a comparison group of children who were baked egg allergic and strictly avoiding baked egg (n = 47). Children who were tolerant to baked egg were 12 times more likely to develop tolerance to regular egg, and developed tolerance faster than children who were baked egg allergic [3]. Interestingly, this study found that once children with baked egg allergy became tolerant to baked egg, they were just as likely to develop tolerance to regular egg as children who were initially tolerant to baked egg. This study also reported that ingestion of baked egg may hasten the resolution of egg allergy.

While these results are intriguing, they must be interpreted in light of the study’s limitations. The comparison group was retrospectively selected and although matched for sex, age and sIgE levels, a comparison of other characteristics between the treatment and comparison group was not presented. If the two groups differed in other characteristics associated with persistent egg allergy, for example SPT wheal size, eczema, severity of previous reactions or other allergies, then the association presented may be biased by not adjusting for these potentially confounding factors. The authors conclude that the ingestion of baked egg accelerates the resolution of egg allergy, however the comparison group’s baked egg allergic status was either allergic or never ingested and avoiding, not those with known baked egg tolerance and avoiding baked egg. Therefore, it may be the underlying phenotype that affects the natural history of egg allergy, rather than the intervention itself.

We have shown previously in a cohort of 12-month old egg allergic infants which were prospectively followed up, that infants who were tolerant to baked egg were 5 times more likely to develop tolerance to raw egg, compared to those who were allergic to baked egg. Among infants with known baked egg tolerance, those who consumed baked egg frequently (≥5 times per month, n = 82) were 3 times more likely to develop tolerance to raw egg compared with those who consumed baked egg infrequently or not at all (n = 29 and n = 6 respectively) [10]. Tey and colleagues were the first to examine the relationship between frequency of baked egg ingestion and rate of decline in egg skin prick test size in egg allergic children [39]. In a retrospective clinical cohort study they found that the mean rate of decline in egg skin prick test size in all children was low (0.7 mm/year). Compared with strict dietary avoidance, frequent consumption of baked egg was not associated with a different rate of decline in egg skin prick test size in egg-allergic children suggesting that low level egg ingestion was not the driver of differential rates of tolerance.

Despite the promising findings of these studies, it is unclear whether the differential development of tolerance is due to the ingestion of baked egg or different clinical phenotypes of egg allergy. Unfortunately the aforementioned studies do not have an adequate control group to address this question, that is, participants with known baked egg tolerance who continue to avoid baked egg. Recruitment of such a control group is likely to be challenging due to the impact of dietary avoidance on nutritional status and quality of life. In the aforementioned HealthNuts study there were 6 infants with confirmed baked egg tolerance who continued to avoid all egg products including baked egg. Their prognosis was worse than the infants who consumed baked egg frequently, however this comparison group of 6 infants is too small to make assertions about the impact of avoiding baked egg in those with known baked egg tolerance. There is currently one ongoing randomised control trials (RCT) assessing the effect of ingesting baked egg to induce tolerance against all egg allergic phenotypes. The CAKE trial (ACTRN12612000173897) in South Australia is recruiting raw egg allergic children who are able to tolerate baked egg, with participants in the intervention group receiving around 30 g baked egg per week. After 6 months participants will cease the product and be re-challenged to raw egg at 7 months. The results of this study will be the first randomised controlled trial to directly assess the effect of baked egg on tolerance induction.

## What is the evidence that eating baked cow’s milk induces tolerance

Research on the impact of dietary baked milk and the natural history of milk allergy is more limited. Similarly to egg, it remains unclear whether regular ingestion of milk in baked goods hastens the development of tolerance against milk allergy. In a prospective study, Nowak-Wegrzyn et al. found that 75 % of milk allergic children could tolerate the baked form of milk in waffles or muffins when challenged [2]. Baked milk tolerant infants were asked to consume products containing baked milk for three months and were rechallenged to raw milk again. Although the results showed that baked milk tolerant infants had lower SPT and higher casein-IgG_4_ than baseline_,_ this was an uncontrolled study and was not compared to a group who did not consume baked milk. In the same cohort, Kim et al. described that baked milk tolerant children were more likely to become tolerant to raw/regular milk than baked milk-reactive children (Odds Ratio 2.8 (95 % Confidence Interval, 4.8–162.7); *p* < 0.01). However, no difference was noted in the milk-specific IgE levels between groups. Due to the absence of suitable comparison groups, it is not clear whether these changes would have naturally occurred over time, whether they are reflective of the underlying milk allergy phenotypes or if they are the results of ingesting milk in baked goods. There are currently no known randomised controlled trials investigating the effect of ingesting milk in baked goods and the resolution of cow milk allergy.

In addition to the potential influence on the development of tolerance, inclusion of egg and milk in its baked form may also have others benefits. It is reasonable to expect that liberation of the diet may boost nutrition, improve the child and family’s quality of life and reduce family anxiety, however no studies have specifically investigated this.

## Conclusion

New methodologies are needed to define egg and milk allergy phenotypes and to assist in determining the likelihood of resolution of the allergy. The allergenicity of major proteins has been difficult to determine due to the complexities surrounding heat treatment and matrix modification on many of the proteins. Further understanding of the molecular basis of the different egg and milk allergy phenotypes may provide valuable opportunities to better differentiate prognosis for allergic infants and children.

Given that dietary restrictions are potentially nutrient deficient and can adversely impact the quality of life of children and their families, it is reasonable to consider liberalizing baked egg and baked milk in the diet of egg allergic and milk allergic children respectively following confirmation of tolerance to these foods in baked form. Both egg and milk allergy have a good prognosis and we have presented observational evidence to support that children who are able to tolerate these food in their baked form are more likely to develop tolerance. However, there is insufficient evidence to conclude that the natural history of egg and milk allergy will be hastened by dietary inclusion of these foods in baked form. The differential natural history on the basis of baked egg and milk tolerance may simply be a marker of a different phenotype of allergy which is less likely to persist.
